# Evaluation of THC-Related Neuropsychiatric Symptoms Among Adults Aged 50 Years and Older: A Systematic Review and Metaregression Analysis

**DOI:** 10.1001/jamanetworkopen.2020.35913

**Published:** 2021-02-02

**Authors:** Latha Velayudhan, Katie Louise McGoohan, Sagnik Bhattacharyya

**Affiliations:** 1 Department of Old Age Psychiatry, Institute of Psychiatry, Psychology, and Neuroscience, Division of Academic Psychiatry, King’s College London, London, United Kingdom; 2 Department of Psychosis, Institute of Psychiatry, Psychology, and Neuroscience, Division of Academic Psychiatry, King’s College London, London, United Kingdom

## Abstract

This systematic review and metaregression analysis estimates the association between the delta-9-tetrahydrocannabinol (THC) dose of cannabinoid-based medicines and neuropsychiatric adverse events among adults aged 50 years and older.

## Introduction

Experimental administration of delta-9-tetrahydrocannabinol (THC), the main psychoactive ingredient in cannabis, but not cannabidiol (CBD), a nonaddictive component, induces transient psychotic symptoms,^1^ and regular use of cannabis high in THC is associated with increased risk of psychotic symptoms or disorders and poor outcomes in those with an established psychotic disorder.^2,3^ This association is well recognized among young people, the age group most often affected by psychosis. Although use of cannabinoid-based medicines (CBMs) is increasing across all age groups, it remains unclear whether THC-containing CBMs also increase the risk of psychotic symptoms in older adults.^4,5^ Hence, we used metaregression analyses to examine any association between THC dose and self-reported neuropsychiatric adverse events (AEs) using data from double-masked, randomized clinical trials (RCTs) investigating CBMs in people aged 50 years or older. We hypothesized that there would be a significant association between THC dose and incidence of neuropsychiatric AEs.

## Methods

We conducted a systematic review of RCTs published until October 31, 2020, undertaken according to the Preferred Reporting Items for Systematic Reviews and Meta-analyses (PRISMA) reporting guideline (eAppendix, eTable, and eFigure in the Supplement), reporting the safety and tolerability of different CBMs (CBD and THC combinations, THC, or its analogues). All-cause and treatment-related AEs were coded according to the Medical Dictionary for Regulatory Activities system organ classes.

Pooled effect sizes (incident rate ratios [IRRs]) were estimated for each AE, and the association of AEs with THC dose (for THC studies) as well as with CBD and THC dose (for CBD and THC combination studies) was examined separately using metaregression analyses under the random-effects model using the restricted maximum-likelihood estimator (metafor package in R version 3.6.3 [R Project for Statistical Computing]), with 2-tailed significance set at *P* < .05. For each broad category of intervention, we combined both parallel-group and crossover RCTs, with the latter treated as parallel-group design.^6^ Studies with more than 1 active treatment group were treated as independent studies.

## Results

Thirty RCTs using THC-only CBMs (15 [50.0%] crossover; 15 [50.0%] parallel-group) analyzed 1417 patients (median [interquartile range {IQR}] age, 59.5 [52.4-67.0] years; median [IQR] percentage men, 52.5% [40.5%-67.8%]; total person-years of THC exposure, 1252.83) in intervention groups and 1210 patients (median [IQR] age, 58.9 [52.0-65.4] years; median [IQR] percentage men, 53.0% [41.3%-71.5%]) in control groups. A total of 24 studies using CBD and THC combinations (5 [20.8%] crossover; 19 [79.2%] parallel-group) analyzed a total of 1917 patients (median [IQR] age, 58.2 [52.3-59.8] years; median [IQR] percentage men, 49.5% [36.0%-56.0%]; total person-years of THC and CBD exposure, 388.56) in intervention groups and 1835 patients (median [IQR] age, 56.0 [53.7-60.3] years; median [IQR] percentage men, 48.0% [35.0%-52.0%]) receiving placebo.

**Figure 1.**
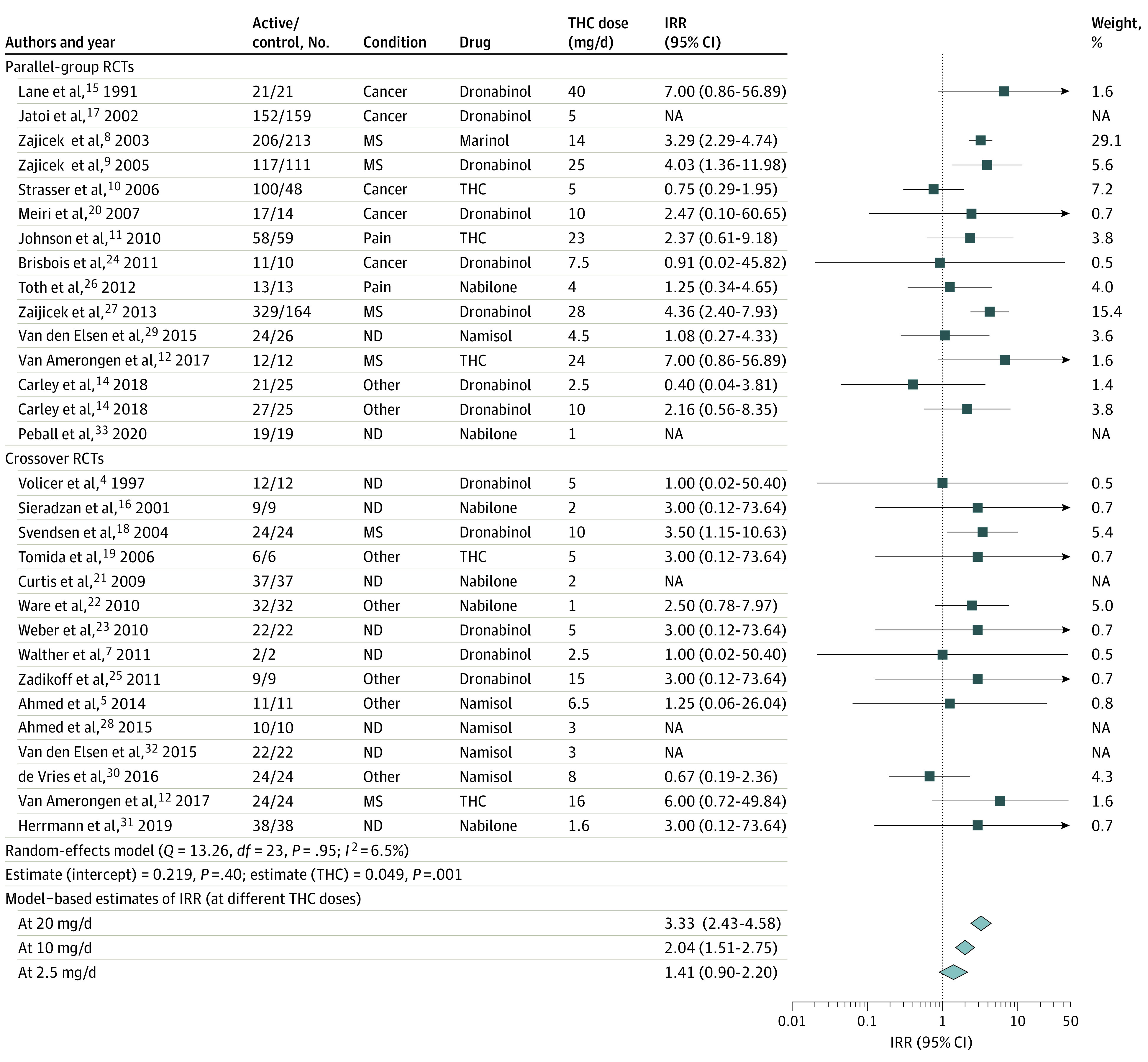
Forest Plot From Metaregression Analysis of Pooled Incident Rate Ratio (IRR) of Dizziness or Lightheadedness Associated With Treatment With Cannabinoid-Based Medicines, With Delta-9-Tetrahydrocannabinol (THC) Dose as a Moderator The disease conditions investigated are listed under the Condition column and were classified into broader subgroups for reporting purposes as neurodegenerative (ND) (ie, Alzheimer disease, Parkinson disease, Huntington disease, amyotrophic lateral sclerosis), multiple sclerosis (MS), pain (ie, neuropathic pain), cancer (ie, cancer- or chemotherapy-related anorexia, pain, or nausea/vomiting), and other (type 2 diabetes, chronic obstructive pulmonary disease, fibromyalgia, raised intraocular pressure, cervical dystonia, healthy, pancreatitis, obstructive sleep apnea, and Levodopa-induced dyskinesia in Parkinson disease). References appear in eReferences in the Supplement. NA indicates not available; RCT, randomized clinical trial.

**Figure 2.**
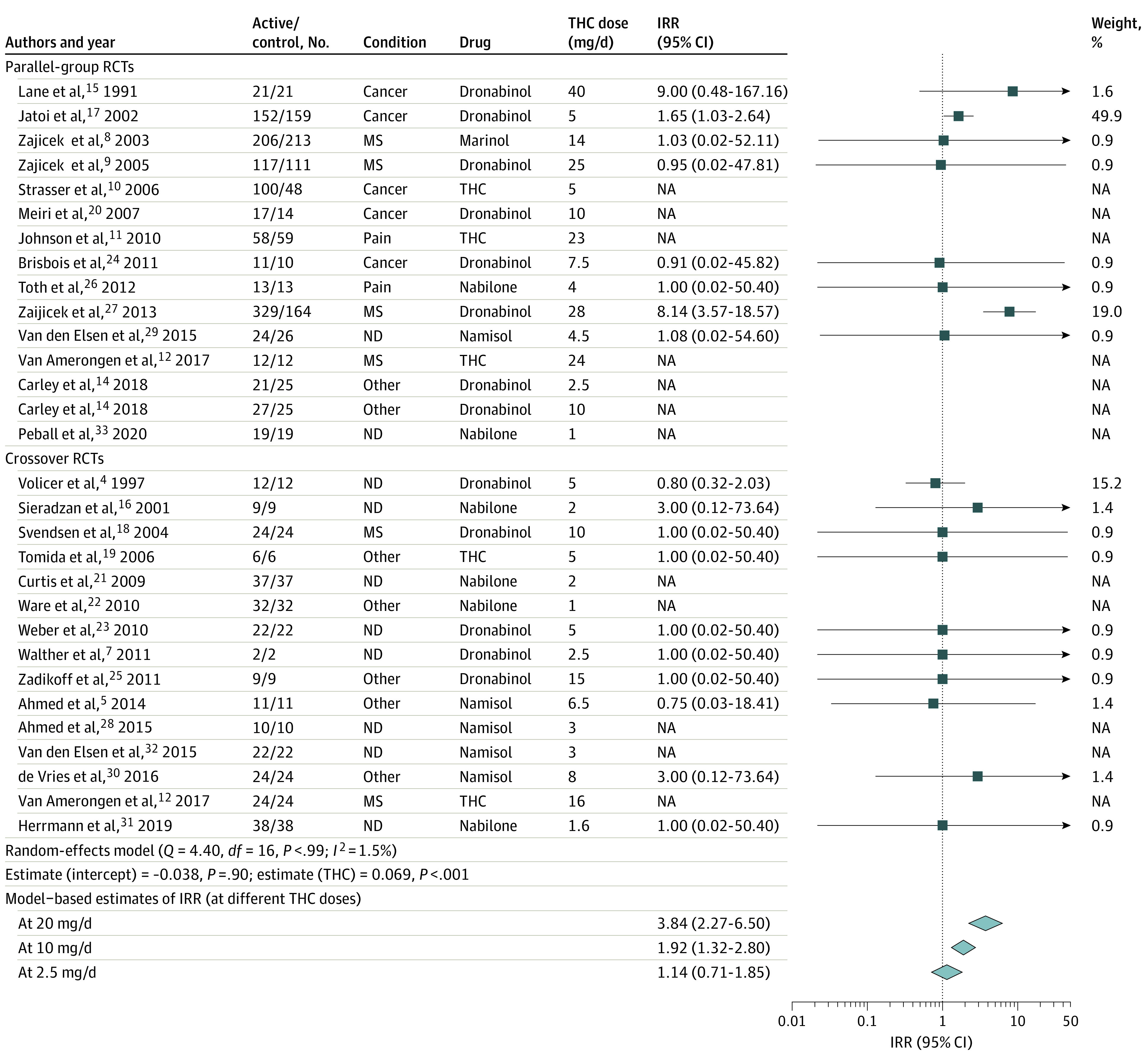
Forest Plot From Metaregression Analysis of Pooled Incident Rate Ratio (IRR) of Thinking/Perception Disorder Associated With Treatment With Cannabinoid-Based Medicines, With Delta-9-Tetrahydrocannabinol (THC) Dose as a Moderator The disease conditions investigated are listed under the Condition column and were classified into broader subgroups for reporting purpose as neurodegenerative (ND) (ie, Alzheimer disease, Parkinson disease, Huntington disease, amyotrophic lateral sclerosis), multiple sclerosis (MS), pain (ie, neuropathic pain), cancer (ie, cancer- or chemotherapy related anorexia, pain, or nausea/vomiting), and other (ie, type 2 diabetes, chronic obstructive pulmonary disease, fibromyalgia, raised intraocular pressure, cervical dystonia, healthy, pancreatitis, obstructive sleep apnea, and Levodopa-induced dyskinesia in Parkinson disease). References appear in eReferences in the Supplement. NA indicates not available; RCT indicates randomized clinical trial.

There was a significant positive association between THC dose and IRR for dizziness or light-headedness (estimate, 0.05; 95% CI, 0.02-0.08; *P* = .001) (Figure 1) and thinking or perception disorder (estimate, 0.07; 95% CI, 0.03-0.11; *P* < .001) (Figure 2) for THC studies, but no association was found with other neuropsychiatric AEs for THC or THC and CBD combination studies. The association with thinking or perception disorder results were associated mainly with 2 studies (eAppendix in the Supplement).

## Discussion

Consistent with our hypothesis, higher THC dose was associated with a higher incidence of thinking or perception disorder and dizziness or light-headedness, but no other neuropsychiatric AEs in RCTs using THC but not THC and CBD combination for a range of nonpsychiatric indications in older adults. Although not diagnosed using standardized assessments, self-reported thinking or perception disorders reflect alterations in thinking and perception typically described under psychotic symptoms and suggest that older adults may also be at risk of psychotomimetic effects from THC. However, this association may be considered tentative based on influence diagnostics. Key limitations of the present analyses are the inability to exclusively focus on older adults or conduct sensitivity analyses in those aged 65 years or older because of limited studies (n = 4); use of self-report rather than structured questionnaires, potentially resulting in underreporting of psychotomimetic effects; and incomplete tolerability reporting in included studies. Given the lack of studies in the population aged 65 years or older, the lack of further AEs in that age group cannot be inferred from our findings. Thus, these results indicate that THC-containing CBMs should be used cautiously in those aged 50 years or older, especially considering that dizziness or light-headedness may increase the risk of falls among older adults.

## Notes

Supplement. **eAppendix. **Supplementary Methods **eReferences.** **eTable. **Characteristics of Included Randomized Clinical Trials **eFigure. **Study Flow Diagram
