# Supplementary material for: Evaluation of THC-Related Neuropsychiatric Symptoms Among Adults Aged 50 Years and Older: A Systematic Review and Metaregression Analysis
Source: JAMA Netw Open. 2021 Feb 2;4(2):e2035913. doi: 10.1001/jamanetworkopen.2020.35913 (PMC7856542; doi:10.1001/jamanetworkopen.2020.35913)
Supplement: Supplement. — eAppendix. Supplementary Methods eReferences. eTable. Characteristics of Included Randomized Clinical Trials eFigure. Study Flow Diagram [file jamanetwopen-e2035913-s001.pdf]

Supplemental Online Content

Velayudhan L, McGoohan KL, Bhattacharyya S. Evaluation of THC-related neuropsychiatric symptoms among adults aged 50 years and older: a systematic review and metaregression analysis. *JAMA Netw Open*. 2021;4(2):e2035913.
doi:10.1001/jamanetworkopen.2020.35913

eAppendix. Supplementary Methods

eReferences.

eTable. Characteristics of Included Randomized Clinical Trials

eFigure. Study Flow Diagram

This supplemental material has been provided by the authors to give readers additional information about their work.

eAppendix. Supplementary Methods

This report is part of a larger systematic review project (also see Velayudhan et al 2020)[1], the protocol for which was pre-registered with the International Prospective Register of Systematic Reviews (PROSPERO) (CRD42019148869). The review was undertaken according to the Preferred Reporting Items for Systematic Reviews and Meta-analyses (PRISMA) (eFigure1) reporting guidelines[2]. Studies were independently assessed by 2 researchers and disagreements resolved through consensus or discussions with a third researcher.

Search strategy

Two categories of search terms were used. For subject groups we used: 'Aged' OR 'frail' OR 'elderly' OR 'older' OR 'aging' OR 'ageing' OR 'geriatric' OR 'dementia' OR 'Parkinson's' OR Alzheimer's' OR 'Huntington's' OR 'demented'. For the intervention, we used: 'Cannabinoids' OR 'cannabinoid' OR 'cannabinol' OR 'cannabidiol' OR 'tetrahydrocannabinol' OR 'THC' OR 'CBD' OR 'Sativex' OR 'nabilone' OR 'dronabinol' OR 'delta-9-tetrahydrocannabinol' OR 'delta-THC' OR 'medical cannabis' OR 'epidiolex'. The existing clinical query 'Therapy/Broad' was used in PubMed to select therapeutic studies. We identified additional studies from the reference lists of included studies and review articles. The search was complemented with information from ClinicalTrials.gov. We also contacted authors of the identified studies to clarify further appropriateness of inclusion if needed.

Study selection

Studies were included if (1) published from 1990 onwards; (2) included older adults (defined as mean age ≥50 years) or reported a distinct subgroup of older adults and provided separate results for this subgroup; and (3) provided data on the safety and tolerability of medical cannabinoids administered by any route, at any dose, for any duration and for any indication. Studies were excluded if they (1) included exclusively younger subjects (mean age <50 years); (2) studied effects of cannabinoids for recreational purposes or failed to provide the dosage of cannabinoids; and (3) were not reported in English language. Here we focus on results from randomised controlled trials (RCTs).

The search strategy identified 4132 citations (Pubmed n = 1305; OVID (Medline, EMBASE and Psychinfo) n = 2041; CINAHL n =786). Adjustment for duplicates left 3688 citations. Of these, 3427 were excluded based on screening of title and abstract. 261 full text articles were retrieved and assessed based on the eligibility criteria. We therefore identified a total of 74 articles regarding safety issues of cannabinoid medications between January 1990 to 31st Oct 2020. Not all published cannabinoid trials provided safety information; we excluded randomized controlled trials[3], because they did not report or quantify adverse events, and one study that used THCV[4]. Limiting our focus to the analysis of randomised trials only left 44 articles to be included in our analysis(Supplement eTable 1a-b). Of these, four studies recruited participants over age ≥ 65 years (n=34; mean age, 72.4 (SD± 4.5)) [5-8].
Five articles studied both THC alone and THC:CBD combinations to compare with placebo [4,9-12]. There was one article that had 2 phases, crossover challenge phase and RCT for same indication [13]. Another article studied the same intervention for two treatment groups compared with separate placebo groups [7]. Two articles used different doses of the same intervention compared with placebo group for each dose [14,15]. This resulted in a final set of 30 RCTs investigating THC (reported as 15 crossover and 15 parallel-arm comparisons) [5,6,8-13,15-34] and 24 studies investigating CBD:THC as treatment (reported as 5 crossover and 19 parallel-arm comparisons) (Supplement eTable 1a-b) [7,9-12,14,35-48].

Rationale for age cut-off:

For this meta-regression analysis, we chose mean age ≥ 50 years as the cut-off as the clinical conditions (diabetes, cancer, neurodegenerative disorders, cancer etc) for which CBMs are often considered afflict people more commonly from around this age. This period of life onwards is also characterised by multi-morbidities, polypharmacy and age-related bodily changes that may affect pharmacokinetics [49] and tolerability of medications. In addition, the age range as well as median and interquartile range of the mean ages of study participants included in the studies that constitute our meta-analysis clearly indicate that people over 65 and 75 years are currently being recruited into studies of CBMs for various indications. However, while there is a larger evidence base of studies with mean age of participants ≥ 50 years this is very modest for studies where all participants are ≥ 65 years (n=3 studies for THC studies and n=1 for THC:CBD study), the typical cut-off age for defining 'elderly'. In light limited power of individual RCTs to unravel patterns of side-effects, the growing use of CBMs in the elderly and the general perception that they are safe to use, it may be particularly important to examine this by synthesizing currently available evidence to help inform about the safety and tolerability profile of CBMs in those aged 50 years and over rather than wait for the evidence base to mature. Therefore, we

© 2021 Velayudhan L et al. *JAMA Network Open.*

have taken the approach to focus on studies with a mean participant age ≥ 50 years which also capture a substantial number of people who are ≥ 65 years. Future attempts at evidence synthesis may be able to focus only on studies of people ≥ 65 years when a sufficient number of studies have accumulated.

Data extraction

All relevant available data for examination of the safety and tolerability of different CBMs (THC:CBD combination or THC or CBD alone) was collected from eligible studies. This was complemented with information from ClinicalTrials.gov and author responses. Data was extracted for study design, participant characteristics, indication, dosage and duration of intervention, all cause and treatment-related AEs and SAEs, AE-related withdrawals and deaths. AEs and SAEs were coded according to the Medical Dictionary for Regulatory Activities (MedDRA) 'system organ classes' (SOC). Data was also extracted for the top 5 (as reported by each study) AEs for each SOC, where available. Data extraction and coding was verified by a medically qualified researcher and discrepancies resolved following discussions with senior researcher. In the present report, we focus on the AEs categorized under the nervous system or psychiatric disorder of MedDRA SOC to investigate their association with dose/s of CBM/s used.

Quality assessment

We used the GRADE (Grading of Recommendations Assessment, Development and Evaluation) criteria to assess the overall quality of evidence and rate risk of bias, publication bias, imprecision, inconsistency, indirectness, and magnitude of effect [50]. We have summarised the GRADE ratings of very low–, low-, moderate-, or high-quality evidence to reflect the extent to which we have confidence in the effect estimates are correct[51]. This was done by one reviewer (KM) and checked by a second reviewer (LV), and disagreements were resolved via discussion with a third reviewer (SB).

Out of all the RCTs included , we judged 30 (56%) trials (THC studies = 21; CBD:THC studies = 9) to be at low risk of bias, 20 (37%) trials (THC studies = 6; CBD:THC studies =14) at unclear risk of bias and four (7%) trials (THC studies =3; CBD:THC studies =1) to have high risk of bias for safety outcome reporting. Overall, 35 trials were judged to be of moderate to high quality (THC studies = 18; CBD:THC studies =17), of which 15 (43%) trials (THC studies = 10; CBD:THC studies =5) reported all AEs.

The formulations used in THC studies were nabilone (6), dronabinol (marinol) (14), THC (3), THC extract spray (2) and Namisol (5). The combination THC-CBD trials used THC:CBD spray (18), and cannabis extract (6).

A broad range of disease conditions/ clinical indications were investigated in these RCTs and included: Alzheimer's disease, Parkinson's disease, Huntington's disease. Amyotrophic lateral sclerosis, Multiple sclerosis, motor neuron disease, neuropathic pain, cancer (cancer or chemotherapy related anorexia, pain or nausea/vomiting), type 2 diabetes mellitus, chronic obstructive pulmonary disease, fibromyalgia, raised intraocular pressure, cervical dystonia, healthy, pancreatitis, obstructive sleep apnoea and Levodopa induced dyskinesia in Parkinson's disease.

Data synthesis and analysis:

We estimated total exposure to active intervention in person-years by first calculating this for each individual study by multiplying the number of subjects in the active intervention arm with the duration of treatment for that arm for each study and then adding up these study-specific values for all studies under each broad category (THC, THC:CBD) of intervention investigated here.

We estimated pooled effect-sizes if there were 2 or more RCTs for each individual neuropsychiatric AE within each broad category of intervention (THC, THC:CBD) under the random-effects model using the restricted maximum-likelihood estimator because of anticipated heterogeneity. For each broad category of intervention, analyses combined both parallel-arm and crossover RCTs, with the latter treated as parallel-arm design [52] for pooled analyses. We estimated incident rate ratio (IRR) for individual AEs. Studies with more than one active treatment arm were treated as independent studies. We combined the data for all conditions for the analysis of AEs.

To test our primary hypothesis, we carried out meta-regression analyses under the random-effects model using the restricted maximum-likelihood estimator to examine the association of individual neuropsychiatric AEs with the dose of THC used in THC studies and separately with the dose of THC and CBD used in THC:CBD studies.

© 2021 Velayudhan L et al. *JAMA Network Open.*

For our primary analysis we focused on all studies with available data where the mean age of study participants was ≥50 years. We also explored the possibility to carry out sensitivity analysis by restricting the analyses to studies where all participants were ≥ 65 years of age. Sensitivity analyses at a different age cut-off of ≥ 65 years was not possible as there was data from fewer studies (3 THC studies and 1 THC:CBD study) than recommended for meta-regression analyses. We investigated heterogeneity using forest plots and the I^2 statistic and report these in Table 1 and forest-plots. We also carried out formal outlier and influence detection diagnostics [53] for the AE of self-reported 'thinking/ perception disorder', which identified two studies as being influential [18,28]. The association between THC dose and 'thinking/ perception disorder' no longer remained significant, though the direction of effect did not change. While outlier/ influence diagnostics are popular in meta-analysis, others have also recommended against their routine use [54], particularly because of challenges in distinguishing true outliers (where the data is erroneous) from large errors in sampling [55]. Instead, they may be seen as a method of sensitivity analysis and inform confidence in findings [53]. In the present case, this is especially important because one of the influential studies [28] had the largest sample size (329 participants in the active intervention arm and 164 in the control intervention arm) as well the longest duration of treatment (~3 years; with only one other study involving 1 year of treatment) in the meta-analytic dataset. Further, we observed that heterogeneity (as indexed by the I2 statistic) decreased from I2=35.73% when the analysis was conducted without THC dose as a moderator (i.e. simple meta-analysis investigating the pooled effect of THC treatment compared to control treatment in the RCTs included on the AE of self-reported 'thinking/ perception disorder') to I2=1.46% when THC dose was included as a moderator, pointing towards the appropriateness of the meta-regression results presented herein. Therefore, these studies have not been excluded but the results presented and discussed with a caveat.

Statistical analyses were performed using the metafor package in R (version 3.6.3) [56].

© 2021 Velayudhan L et al. *JAMA Network Open.*

eReferences.

1. Velayudhan L, McGoohan K, Bhattacharyya S. Safety and tolerability of natural and synthetic cannabinoids in adults aged over 50 years: a systematic review and meta-analysis. *PLoS Medicine.* 2020 (accepted).
2. Moher D, Liberati A, Tetzlaff J, Altman DG, The PG. Preferred Reporting Items for Systematic Reviews and Meta-Analyses: The PRISMA Statement. *PLOS Medicine.* 2009;6(7):e1000097.
3. Selvarajah D, Gandhi R, Emery CJ, Tesfaye S. Randomized placebo-controlled double-blind clinical trial of cannabis-based medicinal product (Sativex) in painful diabetic neuropathy: depression is a major confounding factor. *Diabetes care.* 2010;33(1):128-130.
4. Jadoon KA, Ratcliffe SH, Barrett DA, et al. Efficacy and Safety of Cannabidiol and Tetrahydrocannabivarin on Glycemic and Lipid Parameters in Patients With Type 2 Diabetes: A Randomized, Double-Blind, Placebo-Controlled, Parallel Group Pilot Study. *Diabetes care.* 2016;39(10):1777-1786.
5. Volicer L, Stelly M, Morris J, McLaughlin J, Volicer BJ. Effects of dronabinol on anorexia and disturbed behavior in patients with Alzheimer's disease. *International journal of geriatric psychiatry.* 1997;12(9):913-919.
6. Ahmed AI, van den Elsen GA, Colbers A, et al. Safety and pharmacokinetics of oral delta-9-tetrahydrocannabinol in healthy older subjects: a randomized controlled trial. *European neuropsychopharmacology : the journal of the European College of Neuropsychopharmacology.* 2014;24(9):1475-1482.
7. Pickering EE, Semple SJ, Nazir MS, et al. Cannabinoid effects on ventilation and breathlessness: a pilot study of efficacy and safety. *Chronic respiratory disease.* 2011;8(2):109-118.
8. Walther S, Schupbach B, Seifritz E, Homan P, Strik W. Randomized, controlled crossover trial of dronabinol, 2.5 mg, for agitation in 2 patients with dementia. *Journal of clinical psychopharmacology.* 2011;31(2):256-258.
9. Zajicek J, Fox P, Sanders H, et al. Cannabinoids for treatment of spasticity and other symptoms related to multiple sclerosis (CAMS study): multicentre randomised placebo-controlled trial. *The Lancet.* 2003;362(9395):1517-1526.
10. Zajicek JP, Sanders HP, Wright DE, et al. Cannabinoids in multiple sclerosis (CAMS) study: safety and efficacy data for 12 months follow up. *Journal of neurology, neurosurgery, and psychiatry.* 2005;76(12):1664-1669.
11. Strasser F, Luftner D, Possinger K, et al. Comparison of orally administered cannabis extract and delta-9-tetrahydrocannabinol in treating patients with cancer-related anorexia-cachexia syndrome: a multicenter, phase III, randomized, double-blind, placebo-controlled clinical trial from the Cannabis-In-Cachexia-Study-Group. *Journal of clinical oncology : official journal of the American Society of Clinical Oncology.* 2006;24(21):3394-3400.
12. Johnson JR, Burnell-Nugent M, Lossignol D, Ganae-Motan ED, Potts R, Fallon MT. Multicenter, double-blind, randomized, placebo-controlled, parallel-group study of the efficacy, safety, and tolerability of THC:CBD extract and THC extract in patients with intractable cancer-related pain. *Journal of pain and symptom management.* 2010;39(2):167-179.
13. van Amerongen G, Kanhai K, Baakman AC, et al. Effects on Spasticity and Neuropathic Pain of an Oral Formulation of Delta9-tetrahydrocannabinol in Patients WithProgressive Multiple Sclerosis. *Clinical therapeutics.* 2018;40(9):1467-1482.
14. Portenoy RK, Ganae-Motan ED, Allende S, et al. Nabiximols for opioid-treated cancer patients with poorly-controlled chronic pain: a randomized, placebo-controlled, graded-dose trial. *The journal of pain : official journal of the American Pain Society.* 2012;13(5):438-449.
15. Carley DW, Prasad B, Reid KJ, et al. Pharmacotherapy of Apnea by Cannabimimetic Enhancement, the PACE Clinical Trial: Effects of Dronabinol in Obstructive Sleep Apnea. *Sleep.* 2018;41(1).

16. Lane M, Vogel CL, Ferguson J, et al. Dronabinol and prochlorperazine in combination for treatment of cancer chemotherapy-induced nausea and vomiting. *Journal of pain and symptom management.* 1991;6(6):352-359.
17. Sieradzan KA, Fox SH, Hill M, Dick JP, Crossman AR, Brotchie JM. Cannabinoids reduce levodopa-induced dyskinesia in Parkinson's disease: a pilot study. *Neurology.* 2001;57(11):2108-2111.
18. Jatoi A, Windschitl HE, Loprinzi CL, et al. Dronabinol versus megestrol acetate versus combination therapy for cancer-associated anorexia: a North Central Cancer Treatment Group study. *Journal of clinical oncology : official journal of the American Society of Clinical Oncology.* 2002;20(2):567-573.
19. Svendsen KB, Jensen TS, Bach FW. Does the cannabinoid dronabinol reduce central pain in multiple sclerosis? Randomised double blind placebo controlled crossover trial. *BMJ (Clinical research ed).* 2004;329(7460):253.
20. Tomida I, Azuara-Blanco A, House H, Flint M, Pertwee RG, Robson PJ. Effect of sublingual application of cannabinoids on intraocular pressure: a pilot study. *Journal of glaucoma.* 2006;15(5):349-353.
21. Meiri E, Jhangiani H, Vredenburgh JJ, et al. Efficacy of dronabinol alone and in combination with ondansetron versus ondansetron alone for delayed chemotherapy-induced nausea and vomiting. *Current medical research and opinion.* 2007;23(3):533-543.
22. Curtis A, Mitchell I, Patel S, Ives N, Rickards H. A pilot study using nabilone for symptomatic treatment in Huntington's disease. *Movement disorders : official journal of the Movement Disorder Society.* 2009;24(15):2254-2259.
23. Ware MA, Fitzcharles MA, Joseph L, Shir Y. The effects of nabilone on sleep in fibromyalgia: results of a randomized controlled trial. *Anesthesia and analgesia.* 2010;110(2):604-610.
24. Weber M, Goldman B, Truniger S. Tetrahydrocannabinol (THC) for cramps in amyotrophic lateral sclerosis: a randomised, double-blind crossover trial. *Journal of neurology, neurosurgery, and psychiatry.* 2010;81(10):1135-1140.
25. Brisbois TD, de Kock IH, Watanabe SM, et al. Delta-9-tetrahydrocannabinol may palliate altered chemosensory perception in cancer patients: results of a randomized, double-blind, placebo-controlled pilot trial. *Annals of oncology : official journal of the European Society for Medical Oncology.* 2011;22(9):2086-2093.
26. Zadikoff C, Wadia PM, Miyasaki J, et al. Cannabinoid, CB1 agonists in cervical dystonia: Failure in a phase IIa randomized controlled trial. *Basal Ganglia.* 2011;1(2):91-95.
27. Toth C, Mawani S, Brady S, et al. An enriched-enrolment, randomized withdrawal, flexible-dose, double-blind, placebo-controlled, parallel assignment efficacy study of nabilone as adjuvant in the treatment of diabetic peripheral neuropathic pain. *Pain.* 2012;153(10):2073-2082.
28. Zajicek J, Ball S, Wright D, et al. Effect of dronabinol on progression in progressive multiple sclerosis (CUPID): a randomised, placebo-controlled trial. *The Lancet Neurology.* 2013;12(9):857-865.
29. Ahmed AI, van den Elsen GA, Colbers A, et al. Safety, pharmacodynamics, and pharmacokinetics of multiple oral doses of delta-9-tetrahydrocannabinol in older persons with dementia. *Psychopharmacology.* 2015;232(14):2587-2595.
30. van den Elsen GA, Ahmed AI, Verkes RJ, et al. Tetrahydrocannabinol for neuropsychiatric symptoms in dementia: A randomized controlled trial. *Neurology.* 2015;84(23):2338-2346.
31. de Vries M, Van Rijckevorsel DC, Vissers KC, Wilder-Smith OH, Van Goor H. Single dose delta-9-tetrahydrocannabinol in chronic pancreatitis patients: analgesic efficacy, pharmacokinetics and tolerability. *British journal of clinical pharmacology.* 2016;81(3):525-537.
32. Herrmann N, Ruthirakuhan M, Gallagher D, et al. Randomized Placebo-Controlled Trial of Nabilone for Agitation in Alzheimer's Disease. *The American journal of geriatric psychiatry : official journal of the American Association for Geriatric Psychiatry.* 2019;27(11):1161-1173.

© 2021 Velayudhan L et al. *JAMA Network Open.*

33. van den Elsen GAH, Ahmed AIA, Verkes RJ, Feuth T, van der Marck MA, Olde Rikkert MGM. Tetrahydrocannabinol in Behavioral Disturbances in Dementia: A Crossover Randomized Controlled Trial. *The American journal of geriatric psychiatry : official journal of the American Association for Geriatric Psychiatry.* 2015;23(12):1214-1224.
34. Peball M, Krismer F, Knaus HG, et al. Non-Motor Symptoms in Parkinson's Disease are Reduced by Nabilone. *Annals of neurology.* 2020;88(4):712-722.
35. Carroll CB, Bain P, Teare L, et al. Cannabis for dyskinesia in Parkison disease: A randomized double-blind crossover study. *Neurology.* 2004;63(7):1245-1250.
36. Vaney C, Heinzel-Gutenbrunner M, Jobin P, et al. Efficacy, safety and tolerability of an orally administered cannabis extract in the treatment of spasticity in patients with multiple sclerosis: a randomized, double-blind, placebo-controlled, crossover study. *Multiple sclerosis (Houndmills, Basingstoke, England).* 2004;10(4):417-424.
37. Wade DT, Makela P, Robson P, House H, Bateman C. Do cannabis-based medicinal extracts have general or specific effects on symptoms in multiple sclerosis? A double-blind, randomized, placebo-controlled study on 160 patients. *Multiple sclerosis (Houndmills, Basingstoke, England).* 2004;10(4):434-441.
38. Blake DR, Robson P, Ho M, Jubb RW, McCabe CS. Preliminary assessment of the efficacy, tolerability and safety of a cannabis-based medicine (Sativex) in the treatment of pain caused by rheumatoid arthritis. *Rheumatology (Oxford, England).* 2006;45(1):50-52.
39. Nurmikko TJ, Serpell MG, Hoggart B, Toomey PJ, Morlion BJ, Haines D. Sativex successfully treats neuropathic pain characterised by allodynia: a randomised, double-blind, placebo-controlled clinical trial. *Pain.* 2007;133(1-3):210-220.
40. Duran M, Perez E, Abanades S, et al. Preliminary efficacy and safety of an oromucosal standardized cannabis extract in chemotherapy-induced nausea and vomiting. *British journal of clinical pharmacology.* 2010;70(5):656-663.
41. Notcutt W, Langford R, Davies P, Ratcliffe S, Potts R. A placebo-controlled, parallel-group, randomized withdrawal study of subjects with symptoms of spasticity due to multiple sclerosis who are receiving long-term Sativex(R) (nabiximols). *Multiple sclerosis (Houndmills, Basingstoke, England).* 2012;18(2):219-228.
42. Zajicek JP, Hobart JC, Slade A, Barnes D, Mattison PG. Multiple sclerosis and extract of cannabis: results of the MUSEC trial. *Journal of neurology, neurosurgery, and psychiatry.* 2012;83(11):1125-1132.
43. Lynch ME, Cesar-Rittenberg P, Hohmann AG. A double-blind, placebo-controlled, crossover pilot trial with extension using an oral mucosal cannabinoid extract for treatment of chemotherapy-induced neuropathic pain. *Journal of pain and symptom management.* 2014;47(1):166-173.
44. Serpell M, Ratcliffe S, Hovorka J, et al. A double-blind, randomized, placebo-controlled, parallel group study of THC/CBD spray in peripheral neuropathic pain treatment. *European journal of pain (London, England).* 2014;18(7):999-1012.
45. Fallon MT, Albert Lux E, McQuade R, et al. Sativex oromucosal spray as adjunctive therapy in advanced cancer patients with chronic pain unalleviated by optimized opioid therapy: two double-blind, randomized, placebo-controlled phase 3 studies. *Br J Pain.* 2017;11(3):119-133.
46. Lichtman AH, Lux EA, McQuade R, et al. Results of a Double-Blind, Randomized, Placebo-Controlled Study of Nabiximols Oromucosal Spray as an Adjunctive Therapy in Advanced Cancer Patients with Chronic Uncontrolled Pain. *Journal of pain and symptom management.* 2018;55(2):179-188.e171.
47. Riva N, Mora G, Soraràu G, et al. Safety and efficacy of nabiximols on spasticity symptoms in patients with motor neuron disease (CANALS): a multicentre, double-blind, randomised, placebo-controlled, phase 2 trial. *The Lancet Neurology.* 2019;18(2):155-164.

© 2021 Velayudhan L et al. *JAMA Network Open.*

48. Markovà J, Essner U, Akmaz B, et al. Sativex® as add-on therapy vs. further optimized first-line ANTispastics (SAVANT) in resistant multiple sclerosis spasticity: a double-blind, placebo-controlled randomised clinical trial. *International Journal of Neuroscience.* 2019;129(2):119-128.
49. Singh S, Bajorek B. Pharmacotherapy in the ageing patient: The impact of age per se (A review). *Ageing research reviews.* 2015;24(Pt B):99-110.
50. Guyatt GH, Oxman AD, Vist GE, et al. GRADE: an emerging consensus on rating quality of evidence and strength of recommendations. *BMJ (Clinical research ed).* 2008;336(7650):924-926.
51. Balshem H, Helfand M, Schünemann HJ, et al. GRADE guidelines: 3. Rating the quality of evidence. *Journal of clinical epidemiology.* 2011;64(4):401-406.
52. Elbourne DR, Altman DG, Higgins JP, Curtin F, Worthington HV, Vail A. Meta-analyses involving cross-over trials: methodological issues. *International Journal of Epidemiology.* 2002;31(1):140-149.
53. Viechtbauer W, Cheung MW. Outlier and influence diagnostics for meta-analysis. *Research synthesis methods.* 2010;1(2):112-125.
54. Hunter JE SF. Methods of Meta-Analysis: Correcting Error and Bias in Research Findings (2nd edn). *Sage: Thousand Oaks, CA, .* 2004.
55. Schmidt F. Meta-Analysis: A Constantly Evolving Research Integration Tool. *Organizational Research Methods - ORGAN RES METHODS.* 2007;11:96-113.
56. Viechtbauer W. Conducting Meta-Analyses in R with the metafor Package *Journal of Statistical Software.* 2010;36(3):48.

© 2021 Velayudhan L et al. *JAMA Network Open.*

eTable. Characteristics of Included Randomized Clinical Trials

Table 1a. Characteristics of included randomised controlled trials of THC in older adults (N=30)

Study ID (country)	Study Design (RCT)	THC: Sample included/analysed N Mean age (SD), Male %	Comparator: Sample included/ analysed N Mean age (SD), Male %	Indication	THC classification	Comparator	THC treatment duration, weeks	Calculated daily average THC dose	Overall GRADE rating for study
Ahmed et al. 2014, (Netherlands)	Crossover	12/11<br>72.00 (5), 50	12/11<br>72.00 (5), 50	Healthy older subjects	Namisol	Placebo	.4	6.5mg	Moderate
Ahmed et al. 2015 (Netherlands)	Crossover	10/10<br>77.30 (5.6), 70	10/10<br>77.30 (5.6), 70	Dementia	Namisol	Placebo	2.6	3 mg	Moderate
Brisbois et al. 2011 (Canada)	Parallel-arm	24/11<br>67.00 (10.9), 64	22/10<br>65.50 (8), 50	Cancer patients with chemosensory alterations	Dronabinol	Placebo	2.6	7.5 mg	Low
Carley et al. 2018 (USA) ‡	Parallel-arm	21/21<br>52.70 (7.7), 76	25/25<br>58.80 (6.1), 72	Obstructive Sleep Apnoea	Dronabinol	Placebo	6.0	2.5 mg	Low
Carley et al. 2018 (USA) ‡	Parallel-arm	27/27<br>54.70 (7), 67	25/25<br>58.80 (6.1), 72	Obstructive Sleep Apnoea	Dronabinol	Placebo	6.0	10 mg	Low
Curtis et al. 2009 (UK)	Crossover	44/37<br>52.00 (9.5), 50	44/37<br>52.00 (9.5), 50	Huntington's disease	Nabilone	Placebo	5.0	2 mg	Low
De Vries et al. 2016 (Netherlands)	Crossover	25/24<br>52.00 (NR), 62	25/24<br>52.00 (NR), 62	Chronic pancreatitis	Namisol	Diazepam	.1	8 mg	Moderate
Herrmann et al. 2019, (Canada)	Crossover	39/38<br>87.00 (10), 77	39/38<br>87.00 (10), 77	Alzheimer's disease	Nabilone	Placebo	6.0	1.6 mg	Moderate
Jatoi et al. 2002 (USA)	Parallel-arm	152/152<br>67.00 (10), 66	159/159<br>65.00 (11), 65	Cancer-related anorexia	Dronabinol	Megestrol acetate	8.1	5 mg	Low

© 2021 Velayudhan L et al. *JAMA Network Open.*

Johnson et al. 2010 (UK)[§]	Parallel-arm	58/58 61.30 (12.5), 52	59/59 60.10 (12.3), 54	Patients with cancer-related pain	THC extract spray	Placebo	2.0	23 mg	Moderate
Lane et al. 1991 (USA)	Parallel-arm	21/21 47.0 (20-68) [*,†] 48	21/21 49.0 (22-64) [*,†] 48	Chemotherapy-induced nausea and vomiting	Dronabinol	Prochlorperazine	.9	40 mg	Low
Meiri et al. 2007 (USA)	Parallel-arm	17/17 61.60 (14.2), 53	14/14 57.20 (8.6), 38	Chemotherapy-induced nausea and vomiting	Dronabinol	Placebo	.7	20 mg	Low
Peball et al. 2020 (Austria)	Parallel-arm	19/19 65.4 (7.94), 53	19/19 64.0 (8.04), 74	Parkinson's disease	Nabilone	Placebo	4.0	0.75 mg	Moderate
Sieradzan et al. 2001(UK)	Crossover	9/9 59.00 (NR), 44	9/9 59.00 (NR), 44	Parkinson's disease	Nabilone	Placebo	.1	2 mg	Very low
Strasser et al. 2006 (Germany)[§]	Parallel-arm	100/100 60.00 (12), 54	48/48 62.00 (10), 52	Cancer-related anorexia	THC	Placebo	6.0	5 mg	Low
Svendsen et al. 2004 (Denmark)	Crossover	24/24 50.0 (NR), 42	24/24 50.0 (NR), 42	Multiple sclerosis	Dronabinol	Placebo	3.0	10 mg	Moderate
Tomida et al. 2006 (UK)[§]	Crossover	6/6 55.30 (5), 100	6/6 55.30 (5), 100	Intraocular pressure	THC extract spray	Placebo	.1	5 mg	Low
Toth et al. 2012 (Canada)	Parallel-arm	13/13 60.80 (15.3), 38	13/13 61.60 (14.6), 69	Diabetic peripheral neuropathic pain	Nabilone	Placebo	5.0	4 mg	Low
Van Amerongen et al. 2017, 2 (Netherlands)[‖]	Crossover	24/24 54.30 (8.9), 33	24/24 54.30 (8.9), 33	Multiple sclerosis	THC	Placebo	.1	16 mg	Moderate
Van Amerongen et al. 2017, 1 (Netherlands)[‖]	Parallel-arm	12/12 57.30 (9), 33	12/12 51.40 (8), 33	Multiple sclerosis	THC	Placebo	4.0	28.5 mg	Moderate
Van den Elsen et al. 2015, 1 (Netherlands)	Parallel-arm	24/24 79.00 (8), 46	26/26 78.00 (7), 54	Dementia	Namisol	Placebo	3.0	4.5 mg	Moderate

© 2021 Velayudhan L et al. *JAMA Network Open.*

Van den Elsen et al. 2015, 2 (Netherlands)	Crossover	22/22<br>76.40 (5.3), 68	22/22<br>76.40 (5.3), 68	Dementia	Namisol	Placebo	2.6	3 mg	Moderate
Volicer et al. 1997 (USA)	Crossover	15/12<br>72.70 (4.9), 92	15/12<br>72.70 (4.9), 92	Alzheimer's disease	Dronabinol	Placebo	6.0	5 mg	Very low
Walther et al. 2011 (Switzerland)	Crossover	2/2<br>78.00 (NR), 100	2/2<br>78.00 (NR), 100	Alzheimer's disease	Dronabinol	Placebo	2.0	2.5 mg	Very low
Ware et al. 2010 (Canada)	Crossover	32/32<br>50.00 (11.2), 16	32/32<br>50.00 (11.2), 16	Fibromyalgia	Nabilone	Amitriptyline	2.0	1 mg	Moderate
Weber et al. 2010 (Switzerland)	Crossover	27/22<br>57.00 (12), 74	27/22<br>57.00 (12), 74	Amyotrophic lateral sclerosis patients with cramps	Dronabinol	Placebo	2.0	10 mg	Moderate
Zadikoff et al. 2011 (Canada)	Crossover	9/9<br>60.00 (7), 0	9/9<br>60.00 (7), 0	Cervical dystonia	Dronabinol	Placebo	3.0	15 mg	Low
Zajicek et al. 2003 (UK) §	Parallel-arm	216/206<br>50.00 (8.2), 31	222/213<br>51.00 (7.6), 37	Multiple sclerosis	Dronabinol	Placebo	14.0	25mg	Moderate
Zajicek et al. 2005 (UK) §	Parallel-arm	125/125<br>50.00 (8.2), 31	120/120<br>51.00 (7.6), 37	Multiple sclerosis	Dronabinol	Placebo	52.0	25 mg	Moderate
Zajicek et al. 2013 (UK)	Parallel-arm	332/329<br>52.30 (7.6), 40	166/164<br>52.00 (8.2), 41	Multiple sclerosis	Dronabinol	Placebo	160.0	28 mg	Moderate

*, Median age (range); †, Included as median age for whole study population was ≥50; ‡, Article included more than one dose level; §, Article included more than one cannabinoid intervention; ||, Article included the results of multiple trials; ¶, Article included multiple study groups/indications ; NR, Not recorded.

© 2021 Velayudhan L et al. *JAMA Network Open.*

Table 1b: Characteristics of included randomised controlled trials of CBD:THC in older adults (N=24)

Study ID (country)	Study Design	CBD/THC: Sample included/ analysed N Mean age (SD), Male %	Comparator: Sample included/ analysed N Mean age (SD), Male %	Indication	CBD/THC classification	Comparator	CBD/THC treatment duration, weeks	Calculated daily average CBD/THC dose	GRADE rating
Blake et al. 2006 (UK)	Parallel-arm	31/31<br>60.9 (10.6), 26	27/27<br>64.9 (8.5), 15	Rheumatoid arthritis	THC:CBD spray	Placebo	5.0	14.6mg THC: 13.5mg CBD	Low
Carroll et al. 2004 (UK)	Crossover	19/17<br>67.0 (NR)<br>63	19/17<br>67.0 (NR)<br>63	Levodopa induced dyskinesia in Parkinson's disease	Cannabis extract	Placebo	4.0	10.2mg THC: 5.1mg CBD	Moderate
Duran et al. 2010 (Spain)	Parallel-arm	7/7<br>50 (41-70) *<br>0	9/9<br>50 (34-76) *<br>11	Chemotherapy induced nausea and vomiting	THC:CBD spray	Placebo	.6	13mg THC : 12mg CBD	Moderate
Fallon et al. 2017, 1 (Multicentre) ǁ	Parallel-arm (withdrawal study)	103/103<br>61.4 (10.9), 61	103103<br>61.6 (11.8), 53	Advanced cancer patients with pain	THC:CBD spray	Placebo	5.0	17.6mg THC: 16.3mg CBD	Moderate
Fallon et al. 2017, 2 (Multicentre) ǁ	Parallel-arm	200/199<br>60.0 (11), 53	199/198<br>59.6 (11), 49	Advanced cancer patients with pain	THC:CBD spray	Placebo	5.0	17mg THC: 15.8mg CBD	Moderate
Johnson et al. 2010, (UK) §	Parallel-arm	60/60<br>59.4 (12.1), 55	59/59<br>60.1 (12.3), 54	Patients with cancer-related pain	THC:CBD spray	Placebo	2.0	25mg THC: 23mg CBD	Moderate
Litchman et al. 2018,	Parallel-arm	199/199<br>59.2 (12), 56	198/198<br>60.7 (11.1), 52	Advanced cancer patients with pain	THC:CBD spray	Placebo	5.0	17.3mg THC: 16mg CBD	Moderate

© 2021 Velayudhan L et al. *JAMA Network Open.*

(Multicentre)									
Lynch et al. 2014 (USA)	Crossover	18/16<br>56.0 (10.8), 17	18/16<br>56.0 (10.8), 17	Chemotherapy-induced neuropathic pain	THC:CBD spray	Placebo	6.0	21.6mg THC: 20mg CBD	Low
Markova et al. 2019, (Czech Republic)	Parallel-arm	53/53<br>51.3 (10.2)<br>30	53/53<br>51.3 (10.2)<br>30	Multiple sclerosis	THC:CBD spray	Placebo	12.0	19.7mg THC: 18.3mg CBD	Low
Notcutt et al. 2012 (UK)	Parallel-arm (withdrawal study)	18/18<br>59.7 (9)<br>50	18/18<br>54.4 (10.4)<br>33	Multiple sclerosis	THC:CBD spray	Placebo	4.0	20.8mg THC: 19.3mg CBD	Very low
Nurmikko et al. 2007, (UK)	Parallel-arm	63/63<br>52.4 (15.8), 44	62/62<br>54.3 (15.2), 37	Neuropathic pain	THC:CBD spray	Placebo	5.0	THC 29.7mg: CBD 27.5mg	High
***Pickering et al. 2011, 1 (UK) ¶	Crossover	5/4<br>67.0 (NR), 50	5/4<br>67.0 (NR), 50	COPD	THC:CBD spray	Placebo	.1	4.7mg THC: 4.4mg CBD	Low
Pickering et al. 2011, 2 (UK) ¶	Crossover	6/5<br>58.0 (NR), 80	6/5<br>58.0 (NR), 80	Healthy controls	THC:CBD spray	Placebo	.1	10.3mg THC: 9.5mg CBD	Low
Portenoy et al. 2012, 1 (Multicentre) ‡	Parallel-arm	91/91<br>59.0 (12.3), 49	91/91<br>56.0 (12.2), 48	Cancer patients with chronic pain	THC:CBD spray	Placebo	5.0	10.8mg THC: 10mg CBD	Moderate
Portenoy et al. 2012, 2 (Multicentre) ‡	Parallel-arm	88/87<br>59.0 (13.1), 56	91/91<br>56.0 (12.2), 48	Cancer patients with chronic pain	THC:CBD spray	Placebo	5.0	27mgTHC: 25mg CBD	Moderate

© 2021 Velayudhan L et al. *JAMA Network Open.*

Portenoy et al. 2012, 3 (Multicentre) ‡	Parallel-arm	90/90<br>58.0 (11.2), 53	91/91<br>56.0 (12.2), 48	Cancer patients with chronic pain	THC:CBD spray	Placebo	5.0	43.2mg THC: 40mg CBD	Moderate
Riva et al. 2019 (Italy)	Parallel-arm	30/29<br>58.4 (10.6)<br>62	30/30<br>57.2 (13.8)<br>53	Motor neurone disease	THC:CBD spray	Placebo	6.0	21.6mg THC: 20.0mg CBD	High
Serpell et al. 2014, (UK)	Parallel-arm	128/128<br>57.6 (14.4), 34	118/118<br>57.0 (14.1), 45	Neuropathic pain	THC:CBD spray	Placebo	14.0	24mg THC: 22mg CBD	Moderate
Strasser et al. 2006 (Germany) §	Parallel-arm	95/95<br>61.0 (12), 56	48/48<br>62.0 (10), 52	Cancer-related anorexia	Cannabis extract	Placebo	6.0	5mg THC: 2mg CBD	Moderate
Vaney et al. 2004 (Switzerland)	Crossover	57/50<br>55.0 (10), 49	57/50<br>55.0 (10), 49	Multiple sclerosis	Cannabis extract	Placebo	2.0	27.5mg THC: 9.9mg CBD	Low
Wade et al. 2004 (UK)	Parallel-arm	80/80<br>51.0 (9.4), 41	80/80<br>50.0 (9.3), 35	Multiple sclerosis	THC:CBD spray	Placebo	6.0	40.5mg THC: 37.5mg CBD	Moderate
Zajicek et al. 2003, (UK) §	Parallel-arm	219/211<br>51.0 (7.6), 36	222/213<br>51.0 (7.6), 37	Multiple sclerosis	Cannabis extract	Placebo	14.0	25mg THC: 12.5mg CBD	Moderate
Zajicek et al. 2005, (UK) §	Parallel-arm	138/138<br>51.0 (7.6), 36	120/120<br>51.0 (7.6), 37	Multiple sclerosis	Cannabis extract	Placebo	52.0	25mg THC: 12.5mg CBD	Moderate
Zajicek et al. 2012, (UK)	Parallel-arm	144/143<br>51.9 (7.7), 39	135/134<br>52.0 (7.9), 35	Multiple sclerosis	Cannabis extract	Placebo	12.0	25mg THC: 12.5mg CBD	Moderate

*, Median age (range); †, Included as median age for whole study population was ≥50; ‡, Article included more than one dose level; §, Article included more than one cannabinoid intervention; ||, Article included the results of multiple trials; ¶, Article included multiple study groups/indications ; NR, Not recorded.

© 2021 Velayudhan L et al. *JAMA Network Open.*

eFigure. Study Flow Diagram

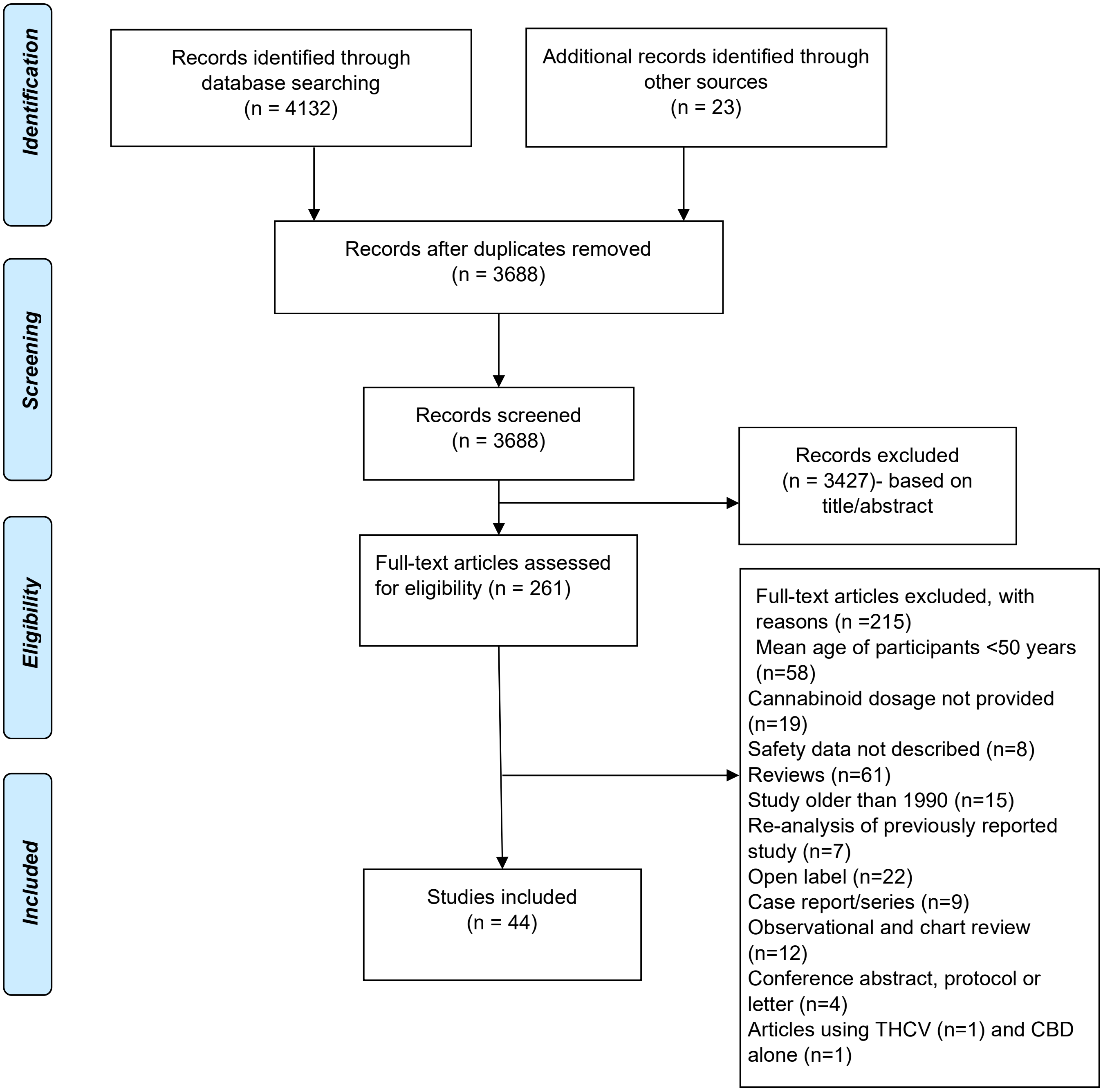

© 2021 Velayudhan L et al. *JAMA Network Open.*
